# Supplementary material for: FastClone is a probabilistic tool for deconvoluting tumor heterogeneity in bulk-sequencing samples
Source: Nat Commun. 2020 Sep 8;11:4469. doi: 10.1038/s41467-020-18169-2 (PMC7478963; doi:10.1038/s41467-020-18169-2)
Supplement: Supplementary file 1 — Supplementary Information [file 41467_2020_18169_MOESM1_ESM.pdf]

Supplementary materials for

Xiao et al.

FastClone is a probabilistic tool for deconvoluting tumor heterogeneity in bulk-sequencing samples

Supplementary Table 1. Symbols and their definitions

Symbol	Definition
ϱ	the prevalence of cells that contain a certain SNV in the tumor sample
ϱ_j	the prevalence of cells that contain the j-th SNV in the tumor sample
β	allele frequency of a certain SNV
β_j	allele frequency of the j-th SNV
$\widehat{\beta}_{jk}$	expected allele frequency of the *k*-th mutation if it is associated with the *j*-th subclone
N_{major}	major copy numbers of CNA
N_{minor}	minor copy numbers of CNA
n_{cell}	total cell number in the tumor
n	total number of SNVs
Z	normalization constant
h	bandwidth for smoothing density estimations
d	number of samples
C	total number of subclones
w_j	the proportion of the *j*-th subclone
L_{jk}	the probability of the *k*-th mutation associated with the *j*-th subclone
λ	log-likelihood of the entire mutation assignment
m_k	observed reads that carry the *k*-th mutation
r_k	total number of reads that cover the locus of the *k*-th mutation and pass the quality filter

Supplementary Table 2. GSEA identifies nine clusters of the 460 variant-associated genes involved in multiple subclone samples

Gene Group #1 Enrichment Score: 2.998054874922734	
Official gene symbol	Gene name
SCUBE2	signal peptide, CUB domain and EGF like domain containing 2(*SCUBE2*)
NELL1	neural EGFL like 1(*NELL1*)
NID1	nidogen 1(*NID1*)
EYS	eyes shut homolog (Drosophila)(*EYS*)
EGFL7	EGF like domain multiple 7(*EGFL7*)
Gene Group #2 Enrichment Score: 2.416966743948818	
Official gene symbol	Gene name
CNTNAP5	contactin associated protein like 5(*CNTNAP5*)
AGTR2	angiotensin II receptor type 2(*AGTR2*)
FSTL4	follistatin like 4(*FSTL4*)
NRSN2	neurensin 2(*NRSN2*)
PKD1L2	polycystin 1 like 2 (gene/pseudogene)(*PKD1L2*)
TSPAN18	tetraspanin 18(*TSPAN18*)
TMEFF2	transmembrane protein with EGF like and two follistatin like domains 2(*TMEFF2*)
DAGLA	diacylglycerol lipase alpha(*DAGLA*)
ROBO2	roundabout guidance receptor 2(*ROBO2*)
ADAM28	ADAM metallopeptidase domain 28(*ADAM28*)
FAT3	FAT atypical cadherin 3(*FAT3*)
B4GALT5	beta-1,4-galactosyltransferase 5(*B4GALT5*)
PCDHB9	protocadherin beta 9(*PCDHB9*)
PCDHB11	protocadherin beta 11(*PCDHB11*)
TRHDE	thyrotropin releasing hormone degrading enzyme(*TRHDE*)
SLCO3A1	solute carrier organic anion transporter family member 3A1(*SLCO3A1*)
MS4A4A	membrane spanning 4-domains A4A(*MS4A4A*)
ADAM23	ADAM metallopeptidase domain 23(*ADAM23*)
FCRL5	Fc receptor like 5(*FCRL5*)
B3GNT9	UDP-GlcNAc:betaGal beta-1,3-N-acetylglucosaminyltransferase 9(*B3GNT9*)
GYPC	glycophorin C (Gerbich blood group)(*GYPC*)
KLHDC7A	kelch domain containing 7A(*KLHDC7A*)
ENPEP	glutamyl aminopeptidase(*ENPEP*)

PIGQ	phosphatidylinositol glycan anchor biosynthesis class Q(*PIGQ*)
NPY2R	neuropeptide Y receptor Y2(*NPY2R*)
HTR2C	5-hydroxytryptamine receptor 2C(*HTR2C*)
SLC4A11	solute carrier family 4 member 11(*SLC4A11*)
SLC6A15	solute carrier family 6 member 15(*SLC6A15*)
OR7C2	olfactory receptor family 7 subfamily C member 2(*OR7C2*)
MAATS1	MYCBP associated and testis expressed 1(*MAATS1*)
LRRN4CL	LRRN4 C-terminal like(*LRRN4CL*)
B3GNT4	UDP-GlcNAc:betaGal beta-1,3-N-acetylglucosaminyltransferase 4(*B3GNT4*)
TMCC1	transmembrane and coiled-coil domain family 1(*TMCC1*)
UNC93A	unc-93 homolog A (C. elegans)(*UNC93A*)
NCAM2	neural cell adhesion molecule 2(*NCAM2*)
SLC5A10	solute carrier family 5 member 10(*SLC5A10*)
PRRG2	proline rich and Gla domain 2(*PRRG2*)
SLC13A4	solute carrier family 13 member 4(*SLC13A4*)
CLSTN2	calsyntenin 2(*CLSTN2*)
PORCN	porcupine homolog (Drosophila)(*PORCN*)
UNC79	unc-79 homolog (C. elegans)(*UNC79*)
PCDH15	protocadherin related 15(*PCDH15*)
DPP6	dipeptidyl peptidase like 6(*DPP6*)
CNTN5	contactin 5(*CNTN5*)
ASTN1	astrotactin 1(*ASTN1*)
SLC16A2	solute carrier family 16 member 2(*SLC16A2*)
PTPRZ1	protein tyrosine phosphatase, receptor type Z1(*PTPRZ1*)
CD300LD	CD300 molecule like family member d(*CD300LD*)
SLC22A25	solute carrier family 22 member 25(*SLC22A25*)
SLC10A2	solute carrier family 10 member 2(*SLC10A2*)
SLC9A3	solute carrier family 9 member A3(*SLC9A3*)
TMEM169	transmembrane protein 169(*TMEM169*)
CHL1	cell adhesion molecule L1 like(*CHL1*)
SLC19A2	solute carrier family 19 member 2(*SLC19A2*)
CDH10	cadherin 10(*CDH10*)
BDKRB2	bradykinin receptor B2(*BDKRB2*)
GRM4	glutamate metabotropic receptor 4(*GRM4*)
RNF130	ring finger protein 130(*RNF130*)
TOR4A	torsin family 4 member A(*TOR4A*)
FUT6	fucosyltransferase 6(*FUT6*)
SLC6A19	solute carrier family 6 member 19(*SLC6A19*)
TEX38	testis expressed 38(*TEX38*)
PTCRA	pre T-cell antigen receptor alpha(*PTCRA*)

MYBPHL	myosin binding protein H like(*MYBPHL*)
OR5AR1	olfactory receptor family 5 subfamily AR member 1 (gene/pseudogene)(*OR5AR1*)
SLC5A4	solute carrier family 5 member 4(*SLC5A4*)
CDCP2	CUB domain containing protein 2(*CDCP2*)
BTNL3	butyrophilin like 3(*BTNL3*)
OR4N2	olfactory receptor family 4 subfamily N member 2(*OR4N2*)
SERINC1	serine incorporator 1(*SERINC1*)
PAQR7	progestin and adipoQ receptor family member 7(*PAQR7*)
OPRK1	opioid receptor kappa 1(*OPRK1*)
OR10X1	olfactory receptor family 10 subfamily X member 1 (gene/pseudogene)(*OR10X1*)
PCDH11X	protocadherin 11 X-linked(*PCDH11X*)
BEST2	bestrophin 2(*BEST2*)
SLC1A2	solute carrier family 1 member 2(*SLC1A2*)
UNC80	unc-80 homolog, NALCN activator(*UNC80*)
MUSK	muscle associated receptor tyrosine kinase(*MUSK*)
ZDHHC11	zinc finger DHHC-type containing 11(*ZDHHC11*)
OR5T2	olfactory receptor family 5 subfamily T member 2(*OR5T2*)
ENPP7	ectonucleotide pyrophosphatase/phosphodiesterase 7(*ENPP7*)
PGAP2	post-GPI attachment to proteins 2(*PGAP2*)
GLT8D2	glycosyltransferase 8 domain containing 2(*GLT8D2*)
FAM171B	family with sequence similarity 171 member B(*FAM171B*)
SYT15	synaptotagmin 15(*SYT15*)
Gene Group #3 Enrichment Score: 1.422264194298178	
Official gene symbol	Gene name
KCNIP1	potassium voltage-gated channel interacting protein 1(*KCNIP1*)
HPCAL1	hippocalcin like 1(*HPCAL1*)
USP32	ubiquitin specific peptidase 32(*USP32*)
CETN1	centrin 1(*CETN1*)
Gene Group #4 Enrichment Score: 1.2841143461144084	
Official gene symbol	Gene name
ERBB4	erb-b2 receptor tyrosine kinase 4(*ERBB4*)
FGFR2	fibroblast growth factor receptor 2(*FGFR2*)
MUSK	muscle associated receptor tyrosine kinase(*MUSK*)
INSR	insulin receptor(*INSR*)

Gene Group #5 Enrichment Score: 1.0384127576755302	
Official gene symbol	Gene name
DYNC1I2	dynein cytoplasmic 1 intermediate chain 2(*DYNC1I2*)
NSMAF	neutral sphingomyelinase activation associated factor(*NSMAF*)
WDR49	WD repeat domain 49(*WDR49*)
WDR91	WD repeat domain 91(*WDR91*)
Gene Group #6 Enrichment Score: 0.9616503618491181	
Official gene symbol	Gene name
MMP13	matrix metallopeptidase 13(*MMP13*)
MMP12	matrix metallopeptidase 12(*MMP12*)
ADAM28	ADAM metallopeptidase domain 28(*ADAM28*)
ADAMTS17	ADAM metallopeptidase with thrombospondin type 1 motif 17(*ADAMTS17*)
Gene Group #7 Enrichment Score: 0.9612591013777856	
Official gene symbol	Gene name
HOXD3	homeobox D3(*HOXD3*)
HOXC4	homeobox C4(*HOXC4*)
PHOX2B	paired like homeobox 2b(*PHOX2B*)
ALX4	ALX homeobox 4(*ALX4*)
LMX1A	LIM homeobox transcription factor 1 alpha(*LMX1A*)
DLX5	distal-less homeobox 5(*DLX5*)
Gene Group #8 Enrichment Score: 0.6245016562904704	
Official gene symbol	Gene name
TRA2A	transformer 2 alpha homolog(*TRA2A*)
RBM47	RNA binding motif protein 47(*RBM47*)
RBM19	RNA binding motif protein 19(*RBM19*)
RBFOX1	RNA binding protein, fox-1 homolog 1(*RBFOX1*)
MTHFSD	methenyltetrahydrofolate synthetase domain containing(*MTHFSD*)
Gene Group #9 Enrichment Score: 0.2180350724391315	
Official gene symbol	Gene name

ST18	ST18, C2H2C-type zinc finger(*ST18*)
GLIS2	GLIS family zinc finger 2(*GLIS2*)
ZKSCAN1	zinc finger with KRAB and SCAN domains 1(*ZKSCAN1*)
ZNF563	zinc finger protein 563(*ZNF563*)
ZNF583	zinc finger protein 583(*ZNF583*)
ZNF415	zinc finger protein 415(*ZNF415*)
PRDM10	PR/SET domain 10(*PRDM10*)
ZNF546	zinc finger protein 546(*ZNF546*)
ZNF335	zinc finger protein 335(*ZNF335*)
ZNF256	zinc finger protein 256(*ZNF256*)
TRPS1	transcriptional repressor GATA binding 1(*TRPS1*)
BNC1	basonuclin 1(*BNC1*)
PATZ1	POZ/BTB and AT hook containing zinc finger 1(*PATZ1*)

Supplementary Table 3. Enrichment in KEGG of the 460 variants associated genes uniquely presented in multi-subclone

Term	PValue	Genes	Fold Enrichment
hsa04930:Type II diabetes mellitus	0.005852402	*HK2, PIK3CA, CACNA1C, ABCC8, INSR, PIK3R2*	5.118303571
hsa04713:Circadian entrainment	0.008081742	*ADCY2, GRIA1, CACNA1I, GUCY1A2, GRIA4, PRKG2, ADCY10, CACNA1C*	3.448120301
hsa04024:cAMP signaling pathway	0.008574512	*PPARA, DRD1, ADCY2, ATP2B4, GRIA1, ATP1A3, PIK3CA, GRIA4, ADCY10, CACNA1C, GLI3, PIK3R2*	2.481601732
hsa04977:Vitamin digestion and absorption	0.015417999	*APOB, CUBN, PLB1, SLC19A2*	7.444805195
hsa04970:Salivary secretion	0.017787335	*BEST2, ADCY2, ATP2B4, GUCY1A2, ATP1A3, PRKG2, MUC5B*	3.332848837
hsa04550:Signaling pathways regulating pluripotency of stem cells	0.020295192	*FGFR2, SMARCAD1, DLX5, FZD1, PIK3CA, JAK1, ACVR1C, KAT6A, PIK3R2*	2.632270408
hsa04022:cGMP-PKG signaling pathway	0.038060074	*ADCY2, ATP2B4, GTF2IRD1, GUCY1A2, ATP1A3, PRKG2, BDKRB2, CACNA1C, INSR*	2.332391501
hsa04611:Platelet activation	0.03841919	*ADCY2, GUCY1A2, PIK3CA, PRKG2, COL5A3, ARHGEF12, ITGB1, PIK3R2*	2.51978022
hsa05146:Amoebiasis	0.04364392	*LAMA3, ACTN4, PIK3CA, COL5A3, ITGAM, CD14, PIK3R2*	2.704009434
hsa04923:Regulation of lipolysis in adipocytes	0.046380273	*ADCY2, PIK3CA, PRKG2, INSR, PIK3R2*	3.655931122
hsa05200:Pathways in cancer	0.053936937	*FGFR2, E2F3, ADCY2, FZD1, EGLN3, BDKRB2, ARHGEF12, ITGB1, GLI3, CTNNA3, TCF7L1, LAMA3, PLEKHG5, PIK3CA, JAK1, PIK3R2*	1.667030171
hsa04512:ECM-receptor interaction	0.059803653	*IBSP, LAMA3, COL6A3, HSPG2, COL5A3, ITGB1*	2.823891626
hsa04974:Protein digestion and absorption	0.062218774	*SLC9A3, COL6A3, ATP1A3, COL12A1, COL5A3, SLC6A19*	2.791801948
hsa04540:Gap junction	0.062218774	*DRD1, ADCY2, GUCY1A2, PRKG2, HTR2C, TUBB4A*	2.791801948
hsa04510:Focal adhesion	0.062816054	*IBSP, LAMA3, ACTN4, RASGRF1, COL6A3, PIK3CA, COL5A3, SHC3, ITGB1, PIK3R2*	1.987690707
hsa04960:Aldosterone-regulated sodium reabsorption	0.067983109	*ATP1A3, PIK3CA, INSR, PIK3R2*	4.1996337
hsa04145:Phagosome	0.072091176	*CTSL, CORO1A, COLEC12, ITGB1, ITGAM, CD14, DYNC1I2, TUBB4A*	2.183809524
hsa05031:Amphetamine addiction	0.075805264	*DRD1, GRIA1, SLC6A3, GRIA4, CACNA1C*	3.102002165
hsa04973:Carbohydrate digestion and absorption	0.081092256	*HK2, ATP1A3, PIK3CA, PIK3R2*	3.899659864
hsa05220:Chronic myeloid leukemia	0.097050662	*E2F3, GAB2, PIK3CA, SHC3, PIK3R2*	2.843501984
hsa04014:Ras signaling pathway	0.098338273	*FGFR2, GAB2, RASGRF1, PIK3CA, RAPGEF5, SHC3, FOXO4, ABL2, INSR, PIK3R2*	1.811788875

hsa04723:Retrograde endocannabinoid signaling	0.098513943	*SLC32A1, DAGLA, ADCY2, GRIA1, GRIA4, CACNA1C*	2.432461103

Supplementary Table 4. GO enrichment of the 460 variants associated genes uniquely presented in multi-subclone

Term	p-value	Genes	Fold Enrichment
GO:0005509~calcium ion binding	3.26E-04	*SYT4, CLSTN2, NELL1, FSTL4, CETN1, SYT7, PCDHB11, KCNIP1, PKD1L2, FAT3, PRRG2, USP32, PCDHB9, MICU1, HPCAL1, CUBN, ACTN4, EGFL7, SCUBE2, PCDH11X, FBN1, HSPG2, NID1, PCDH15, F7, MMP13, MMP12, EYS, WDR49, STAB1, ANXA13, SYT15, NCAN, CDH10, CSN2*	1.91375048
GO:0015293~symporter activity	0.00336821	*SLC32A1, SLC16A2, SLC1A2, SLC5A4, SLC4A11, SLC6A3, SLC6A15, SLC13A4, SLC10A2, SLC6A19, SLC5A10*	3.03697183
GO:0008017~microtubule binding	0.01026958	*FMN1, KIF1C, ARHGEF2, KIF5A, CRYAB, MAP2, CLIP2, CETN1, LRRK2, NME8, SPAST, SGIP1, KIF20A*	2.32721046
GO:0008324~cation transmembrane transporter activity	0.02525162	*KCNC2, SHROOM2, SLC5A4, SLC9A3, SLC6A3, ANO1, KCNA4, KCNIP1, PKD1L2, SLC32A1, SLC1A2, ATP2B4, SCN5A, SCN10A, CACNA1I, ATP1A3, SLC6A15, SLC10A2, SLC6A19, SLC4A11, LOXHD1, SCN11A, SLC13A4, CACNA1C, ABCC8*	1.59109357
GO:0005216~ion channel activity	0.04717185	*KCNC2, SHROOM2, ANO1, CACNA1I, KCNA4, GRIA4, KCNIP1, PKD1L2, BEST2, SLC4A11, LOXHD1, GRIA1, CLIC5, SCN11A, CACNA1C, SCN5A, ABCC8, SCN10A*	1.64494596
GO:0051015~actin filament binding	0.05141841	*CORO1A, SHROOM2, ACTN4, SVIL, MYO16, TMOD4, ABL2, CTNNA3*	2.37603306
GO:0022838~substrate-specific channel activity	0.06179453	*KCNC2, SHROOM2, ANO1, CACNA1I, KCNA4, GRIA4, KCNIP1, PKD1L2, BEST2, SLC4A11, LOXHD1, GRIA1, CLIC5, SCN11A, CACNA1C, SCN5A, ABCC8, SCN10A*	1.58580184
GO:0005524~ATP binding	0.07105869	*PRKAG3, CDK19, FGFR2, SMARCAD1, BTAF1, H1FNT, ADCY2, NARS, ERBB4, TTLL9, HK2, CASK, TOR4A, TTK, PRKG2, BMS1, ABCA6, ACVR1C, ATAD3C, CHD9, MUSK, ATP2B4, MYO15A, VPS4A, PIK3CA, ADCY10, INSR, NSF, TRIP13, ABCE1, OBSCN, MKI67, KIF5A, ALPK2, SARS, MYO1G, ATP1A3, KIF1C, NEK8, MYO16, JAK1, SLFN11, LRRK2, ABCC8, ABL2, SPAST, NLRP10, KIF20A*	1.25874126
GO:0050816~phosphothreonine binding	0.07435652	*FBXW7, NEDD4*	26.1363636
GO:0017075~syntaxin-1 binding	0.07545261	*STXBP3, LRRK2, NSF*	6.53409091
GO:0046943~carboxylic acid transmembrane transporter activity	0.07781724	*SLC32A1, SLC16A2, SLC1A2, SERINC1, SLC6A15, SLC10A2, SLC6A19*	2.3455711
GO:0030554~adenyl nucleotide binding	0.07811479	*CDK19, PRKAG3, ADCY2, NARS, TTLL9, CASK, TTK, PRKG2, ACVR1C, ATAD3C, ATP2B4, BAG3, PIK3CA, VPS4A, ADCY10, INSR, NSF, ABCE1, KIF5A, SARS, KIF1C, NEK8, LRRK2, SPAST, SMARCAD1, FGFR2, BTAF1, H1FNT, ERBB4, HK2, TOR4A, BMS1, ABCA6, CHD9, MUSK, MYO15A, TRIP13, OBSCN, MKI67, ALPK2, ATP1A3, MYO1G, MYO16, JAK1, SLFN11, ABCC8, ABL2, NLRP10, KIF20A*	1.24579943
GO:0001077~transcriptional activator activity, RNA	0.07977679	*PHOX2B, PPARA, ELF3, GLIS2, DLX5, PATZ1, NEUROG3, IRF4, GLI3, FOXI1, NFIB*	1.82733051

polymerase II core promoter proximal region sequence-specific binding			
GO:0022836~gated channel activity	0.08457928	*KCNC2, SHROOM2, GRIA1, CLIC5, ANO1, CACNA1I, KCNA4, SCN11A, GRIA4, CACNA1C, KCNIP1, ABCC8, SCN5A, SCN10A*	1.6433043
GO:0032559~adenyl ribonucleotide binding	0.09626907	*PRKAG3, CDK19, FGFR2, SMARCAD1, BTAF1, H1FNT, ADCY2, NARS, ERBB4, TTLL9, HK2, CASK, TOR4A, TTK, PRKG2, BMS1, ABCA6, ACVR1C, ATAD3C, CHD9, MUSK, ATP2B4, MYO15A, VPS4A, PIK3CA, ADCY10, INSR, NSF, TRIP13, ABCE1, OBSCN, MKI67, KIF5A, ALPK2, SARS, MYO1G, ATP1A3, KIF1C, NEK8, MYO16, JAK1, SLFN11, LRRK2, ABCC8, ABL2, SPAST, NLRP10, KIF20A*	1.22914316
GO:0001847~opsonin receptor activity	0.09789633	*CR1, CD14*	19.6022727

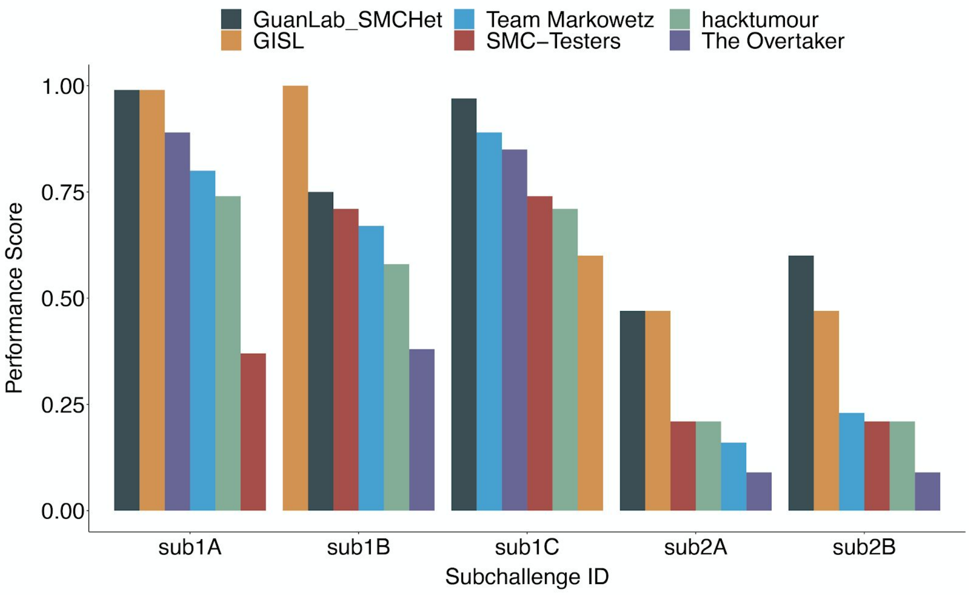

Supplementary Figure 1. Performance comparison among submissions. Median performance scores of different algorithms in each sub-challenge. Sub1A predicts purity of the tumor. Sub1B predicts the number of subclones. Sub1C predicts subclone proportions. Sub2A and Sub2B predict mutation assignments to subclones from different perspectives. Sub2A predicts the assignments of each SNV to subclone, and Sub2B predicts the probabilistic clustering of SNVs. SMC-Tester is the PhyloWGS baseline.

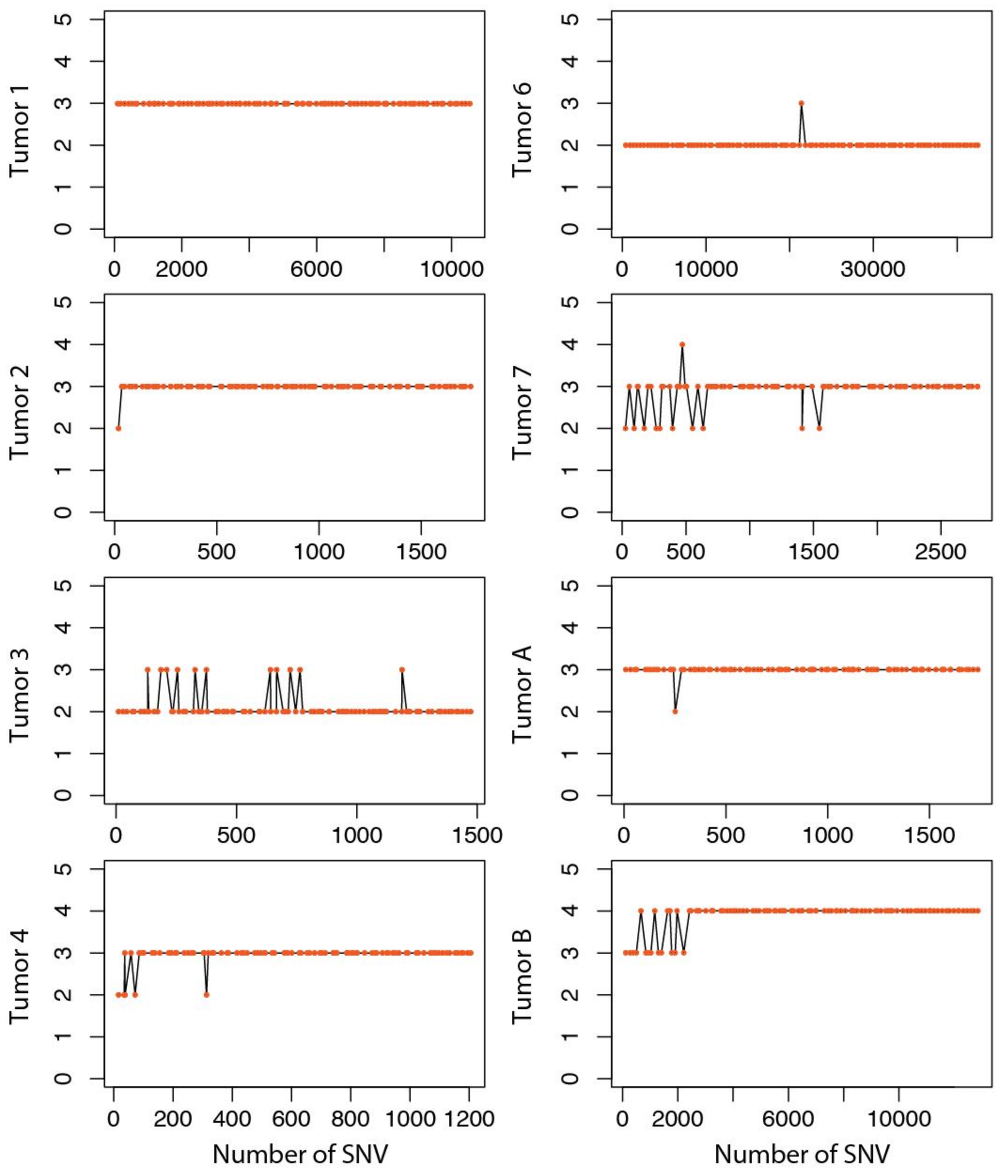

Supplementary Figure 2. Number of subclones predicted by FastClone is mostly robust across different numbers of SNVs. The x axis shows the number of SNVs randomly sampled from the tumor data, and the y axis shows the predicted number of subclones in the tumors. Red dots indicate sampling points.

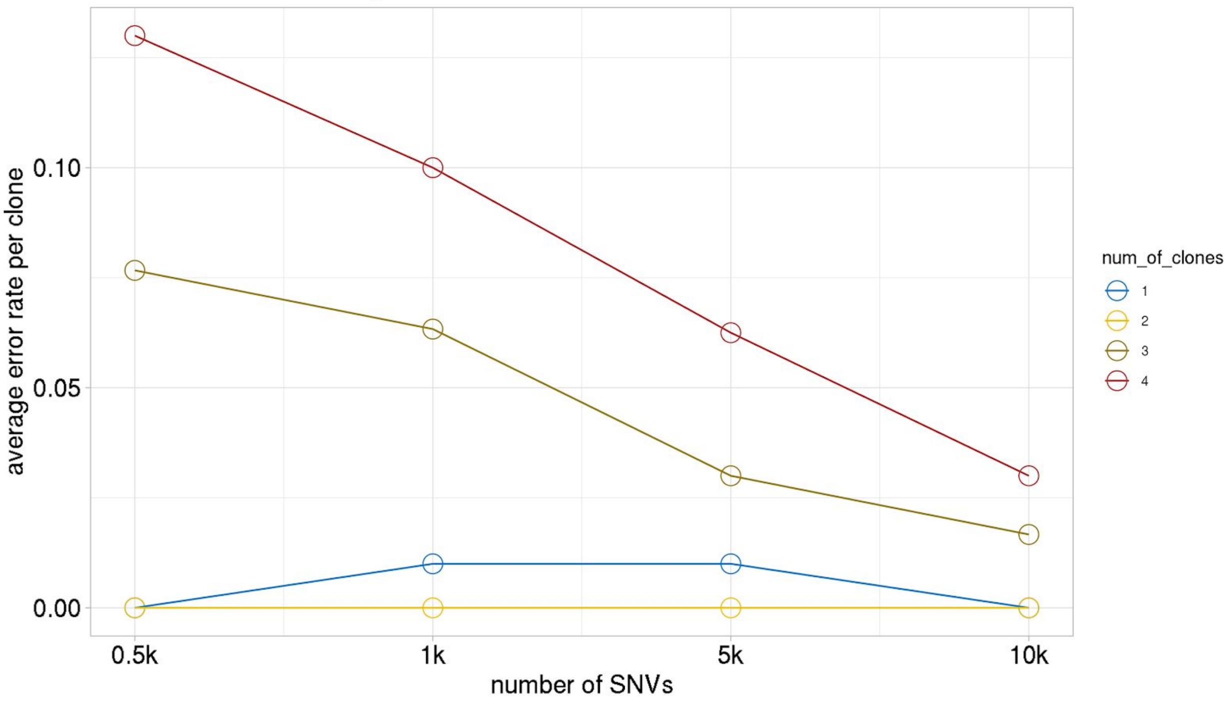

Supplementary Figure 3. FastClone's performance on different numbers of subclones. We simulated ϱ distributions with different numbers of subclones and different numbers of SNVs from hypergeometric distribution. The ϱ values were sampled from a uniform distribution between 0 and 1. For each case, we performed 100 simulations and used FastClone to predict ϱ values. Then the average error rate per subclone was calculated. As the number of SNVs increased, the error rate dropped and approached zero.

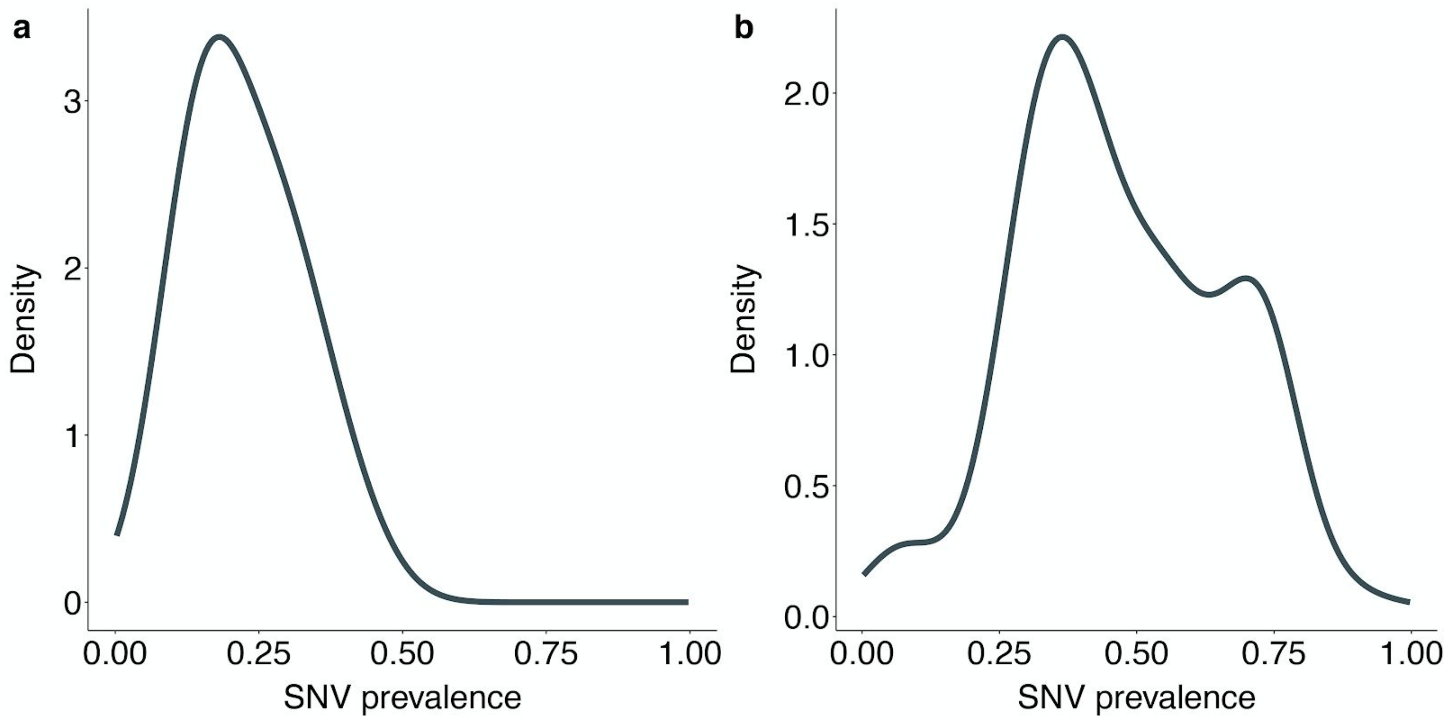

Supplementary Figure 4. FastClone determines the number of subclones based on local peaks in the distribution. This figure shows the density distribution by computing the kernel density estimation of the two primary tumor samples that were obtained from CP08 sample. **(a)** The distribution of T1 primary tumor sample in CP08 suggests there is one subclone in CP08 T1. **(b)** The distribution of T2 primary tumor sample in CP08 suggests there are two subclones in CP08 T2, and the second peak was removed by FastClone based on our model's adjustment rule that the prevalence of a subclone should not surpass tumor purity.

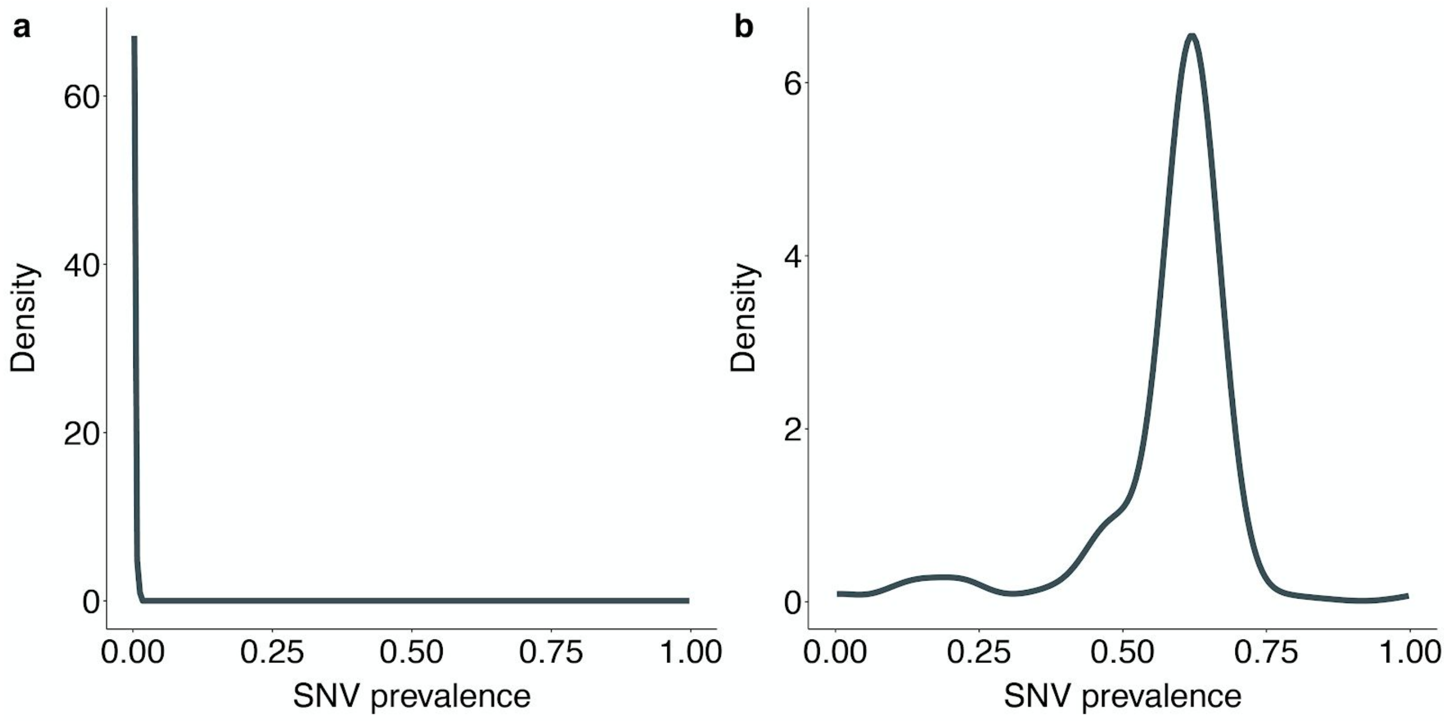

Supplementary Figure 5. The kernel density estimation of the two primary tumor samples obtained from CP11 sample. (a) This is the distribution of T1 primary tumor sample in CP11, as we mentioned in the paper that deep sequencing failed for CP11 primary tumor sample, so it turns out there is one subclone whose proportion is close to 0. (b) The distribution of T2 primary tumor sample in CP11 suggests there are two subclones in CP11 T2.

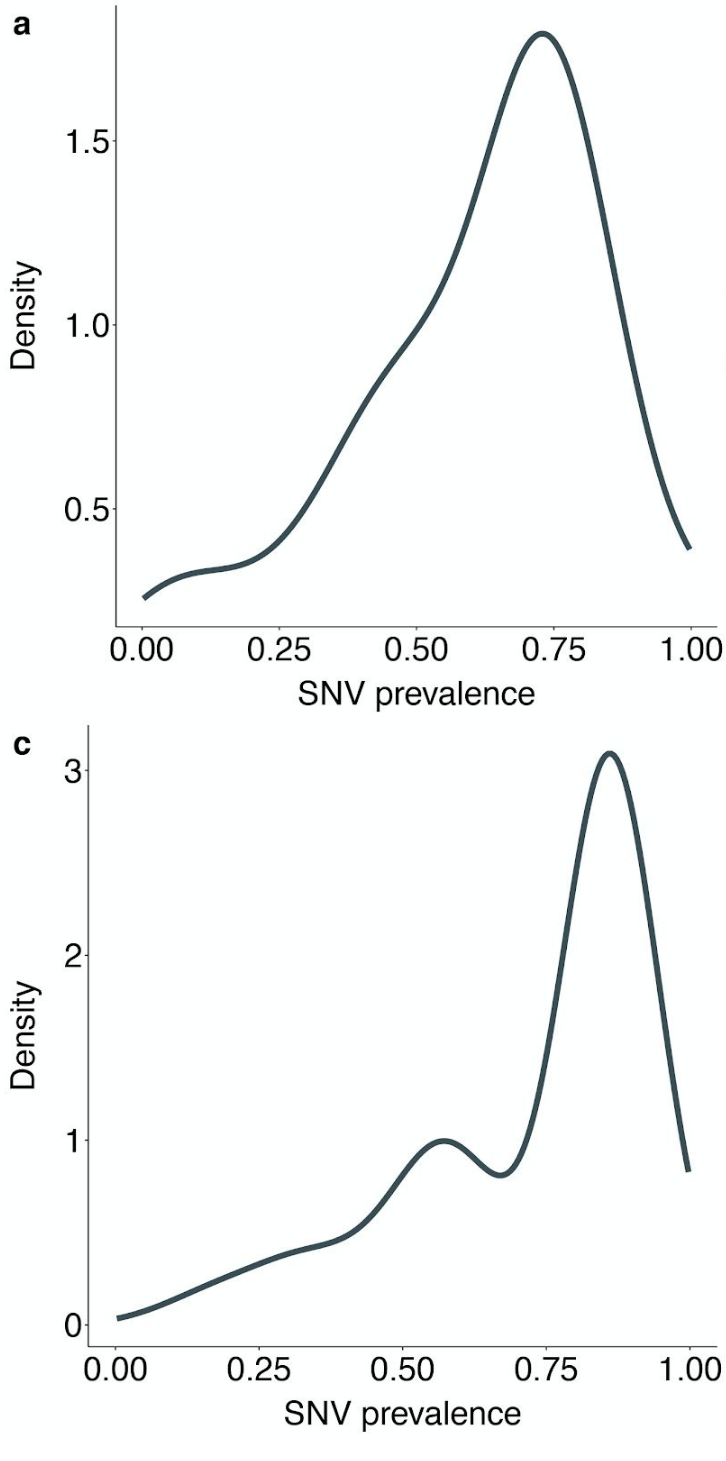

Supplementary Figure 6. The kernel density estimation of the three primary tumor samples from CP14 sample. (a) The distribution of T1 primary tumor sample in CP14 suggests there is one subclone in CP14 T1. (b) The distribution of T2 primary tumor sample in CP14 suggests there are two subclones in CP14 T2. (c) The distribution of T3 primary tumor sample in CP14 suggests there are two subclones in CP14 T3.

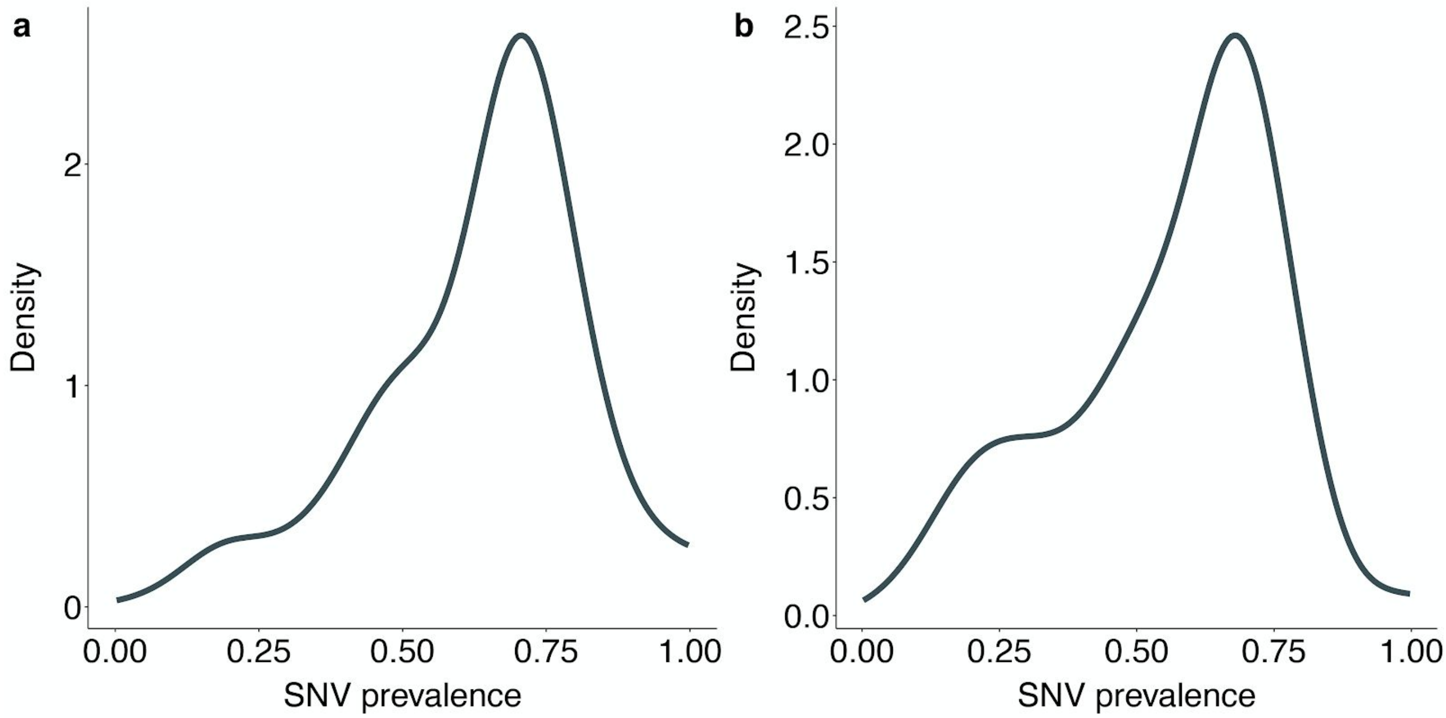

Supplementary Figure 7. The kernel density estimation of the two primary tumor samples obtained from CP15 sample. (a) The distribution of T1 primary tumor sample in CP15 suggests there is one subclone in CP15 T1. (b) The distribution of T2 primary tumor sample in CP15 suggests there is one subclone in CP15 T2.

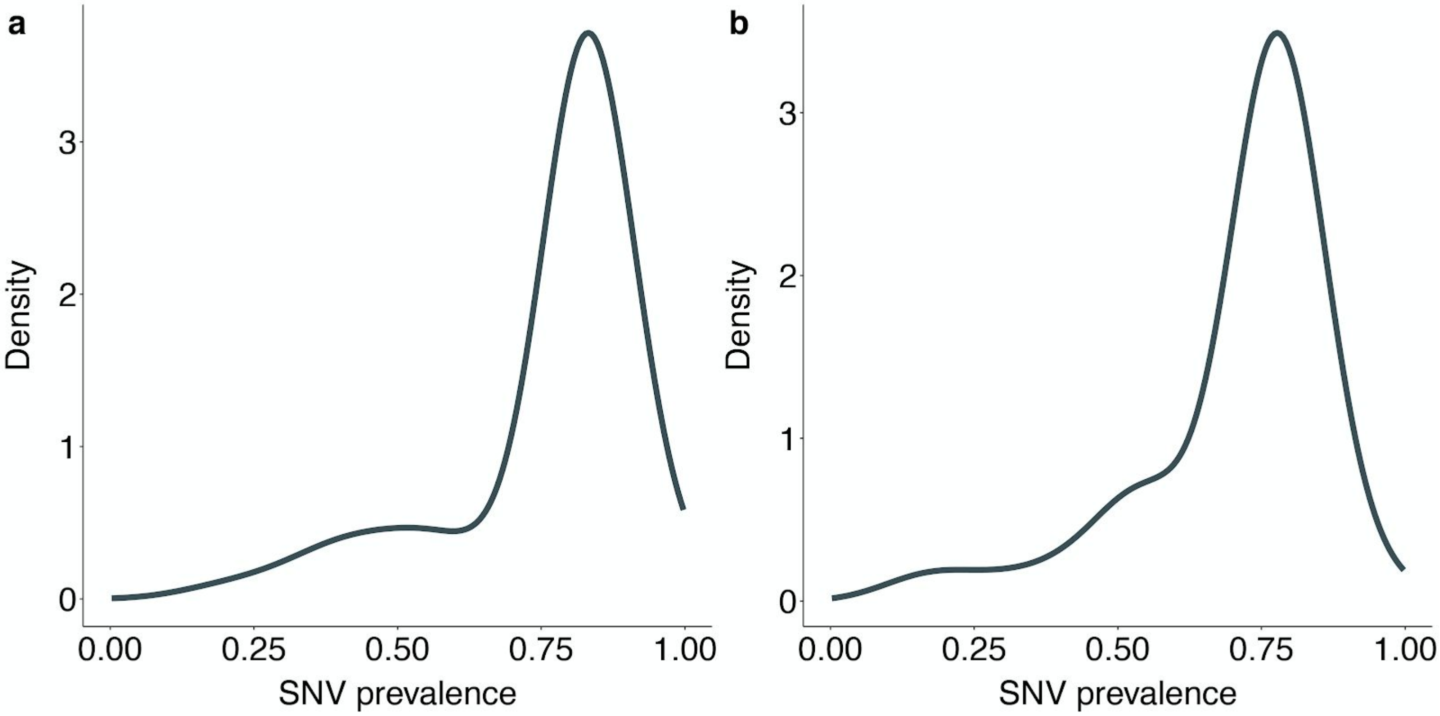

Supplementary Figure 8. The kernel density estimation of the two primary tumor samples obtained from CP17 sample. (a) The distribution of T1 primary tumor sample in CP17 suggests there are two subclones in CP17 T1. (b) The distribution of T2 primary tumor sample in CP17 suggests there are two subclones in CP17 T2.

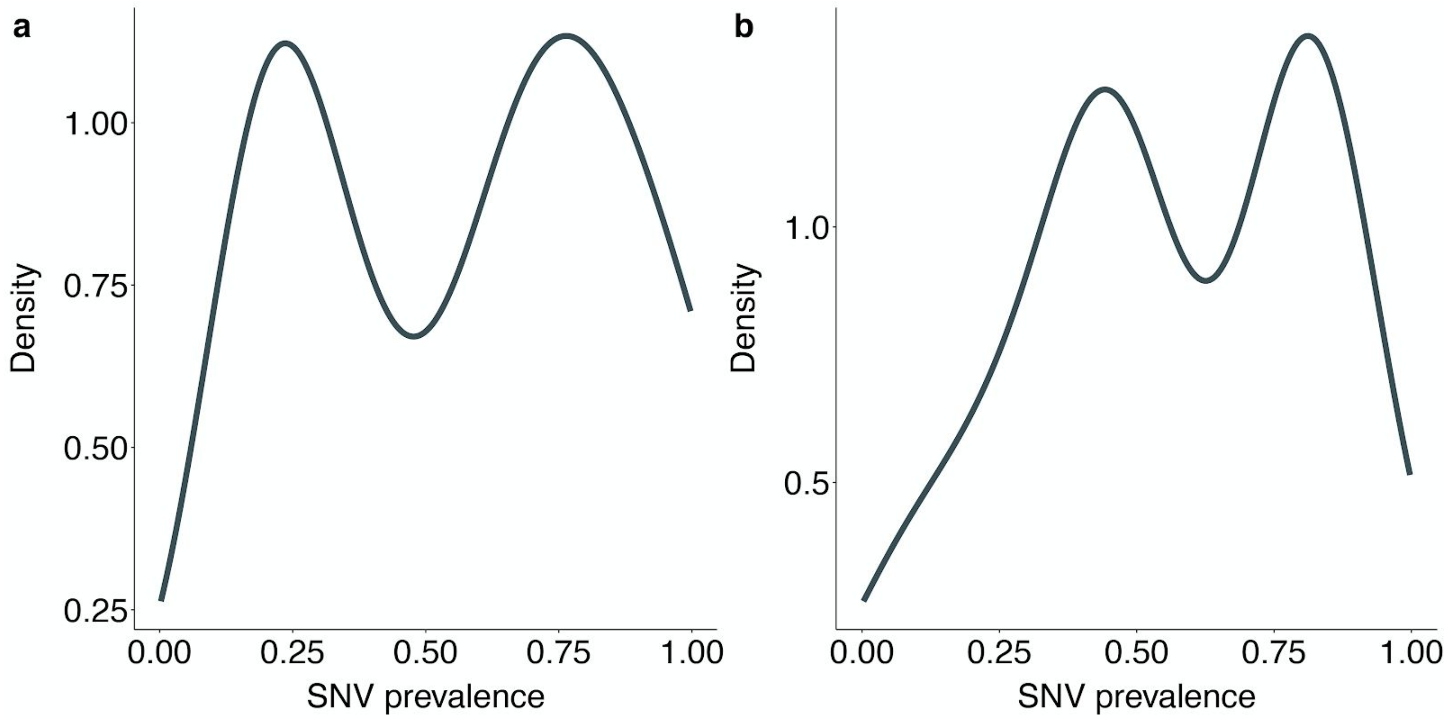

Supplementary Figure 9. The kernel density estimation of the two primary tumor samples obtained from CP18 sample. (a) The distribution of T1 primary tumor sample in CP18 suggests there are two subclones in CP18 T1. FastClone actually added one more peak based on our model's adjustment rule, which means the final result was three subclones. (b) The distribution of T2 primary tumor sample in CP18 suggests there are two subclones in CP18 T2.

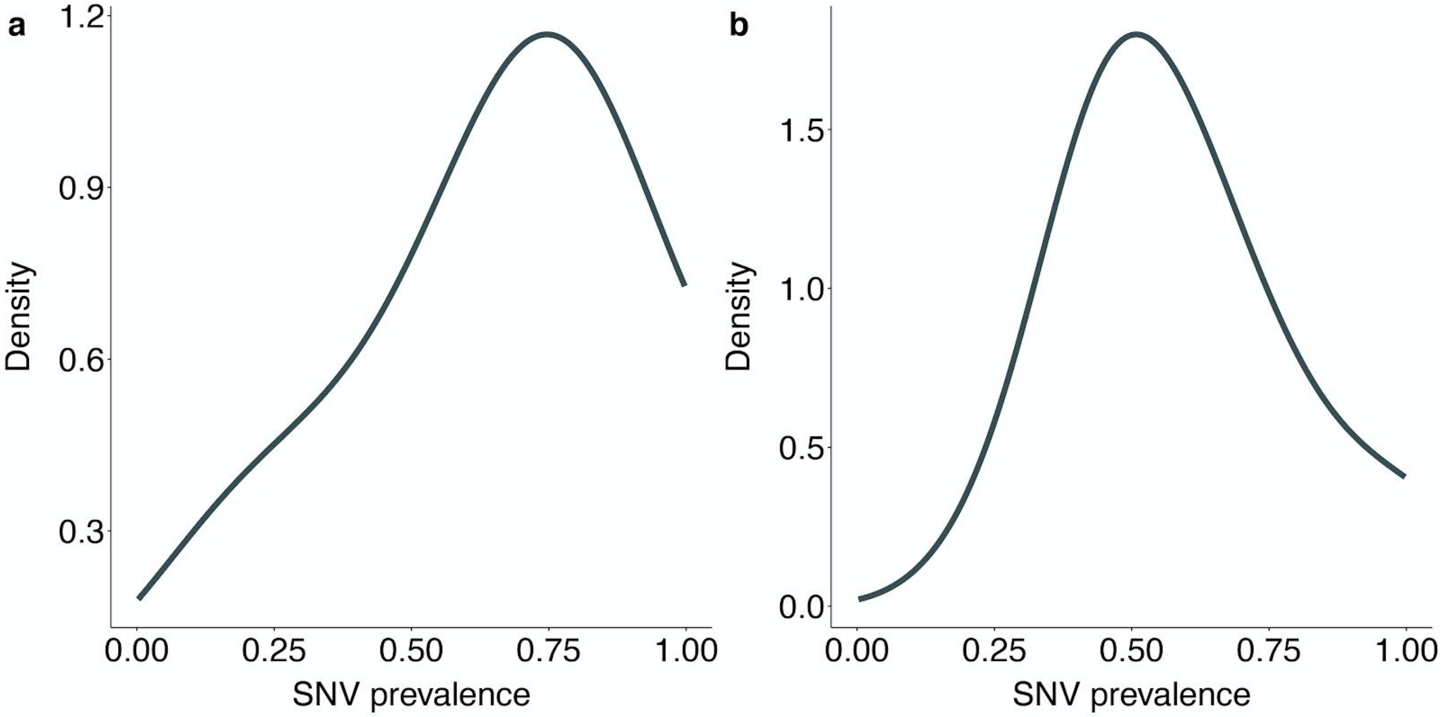

Supplementary Figure 10. The kernel density estimation of the two primary tumor samples from CP19 sample. (a) The distribution of T1 primary tumor sample in CP19 suggests there is one subclone in CP19 T1. (b) The distribution of T2 primary tumor sample in CP19 suggests there is one subclone in CP19 T2.

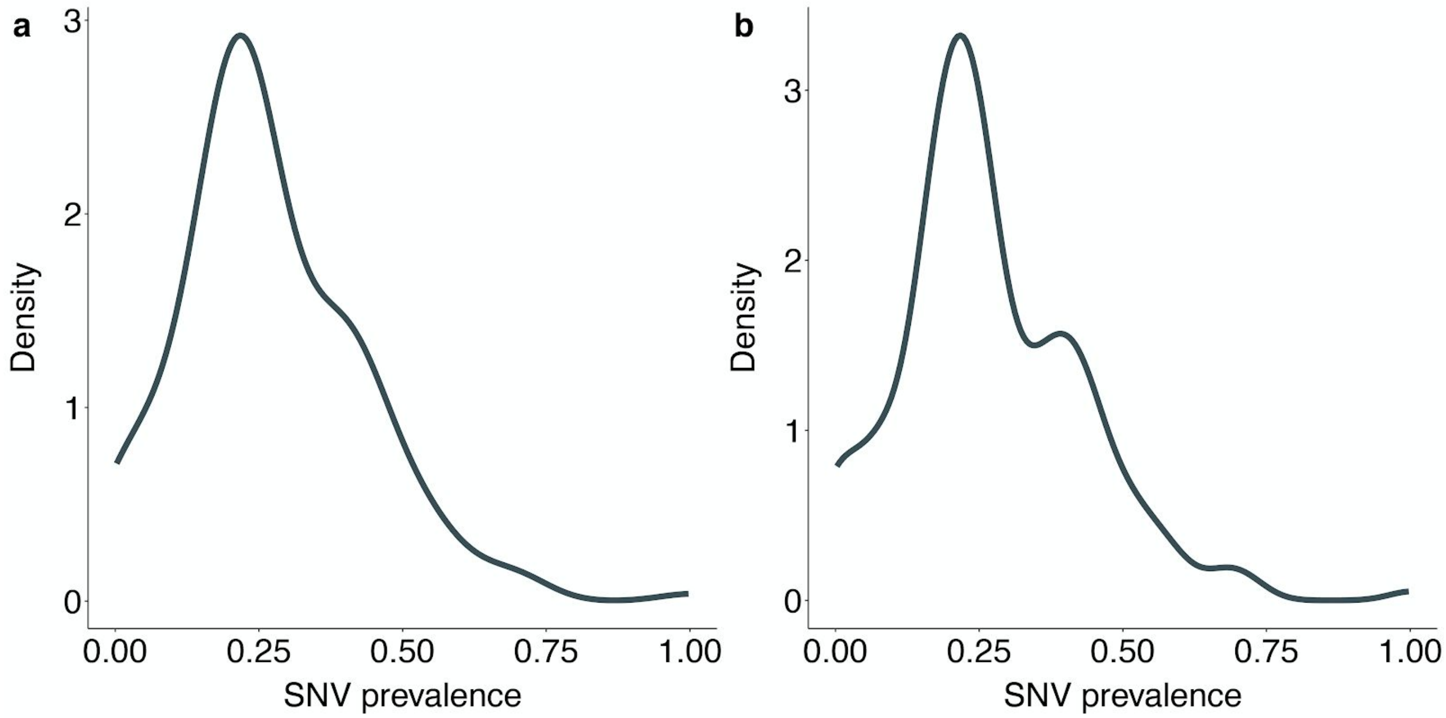

Supplementary Figure 11. Adjustment to mixed Gaussian distributions increases subclone numbers FastClone gives. There are some situations where subclones are not apparent enough to be caught by FastClone. Then, an appropriate adjustment to FastClone is necessary. At the point that FastClone calculates the kernel density estimation of the input list of tumor cells' proportions, we can manually set up the value of covariance factor instead of using the default value. The regulation can help us to detect more subclones, when FastClone misses some tiny subclones that have relatively low proportions. From the example, we can see that when we change the covariance factor of the kde, the number of peaks increases to three from one.

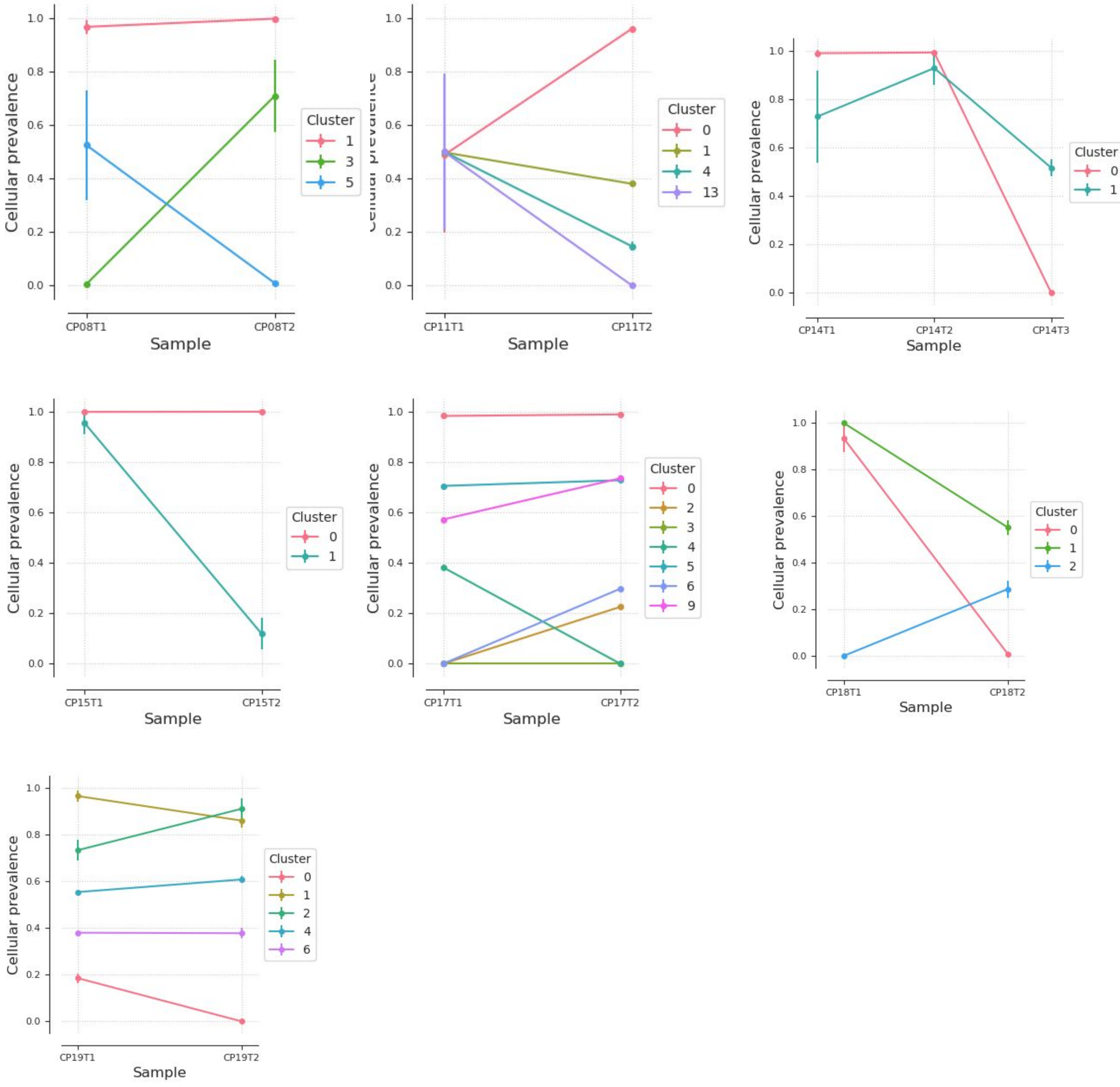

Supplementary Figure 12. PyClone's results of the number of subclones for each primary tumor sample.

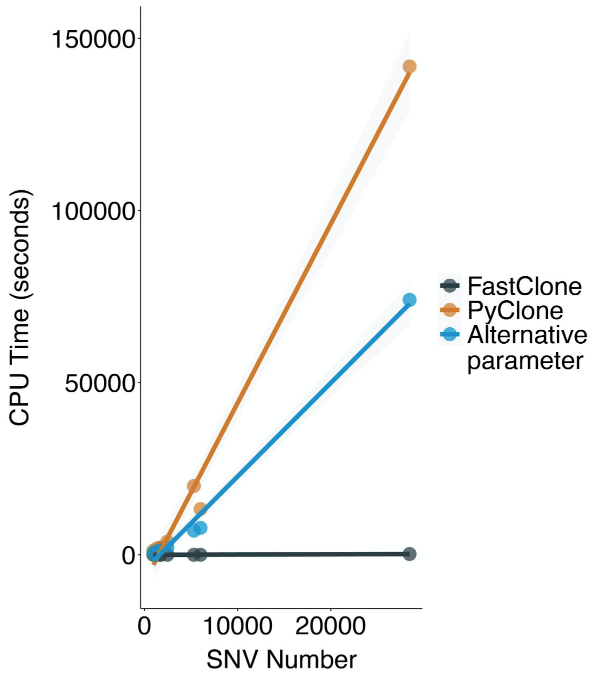

Supplementary Figure 13. The correlation between SNV number and processing time. FastClone, PyClone, and PyClone with an alternative Gamma precision parameter are compared on 8 simulated data from DREAM challenge.

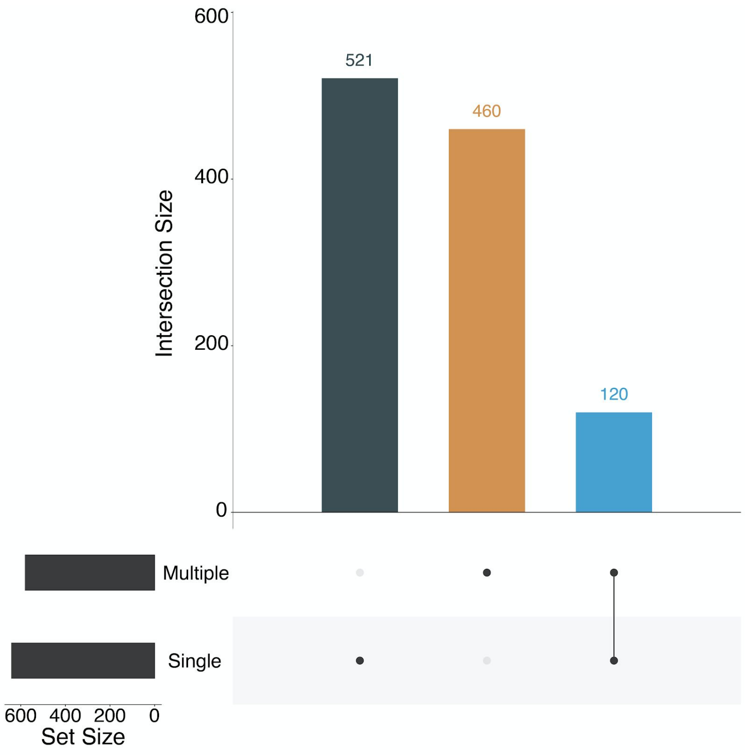

Supplementary Figure 14. Statistics of shared variants and unique variants. The right bar represents the variants that occur in both multiple subclone samples and single subclone samples, the middle bar represents the variants that occur only in multiple subclone samples, and the left bar represents the variants that occur only in single subclone samples.

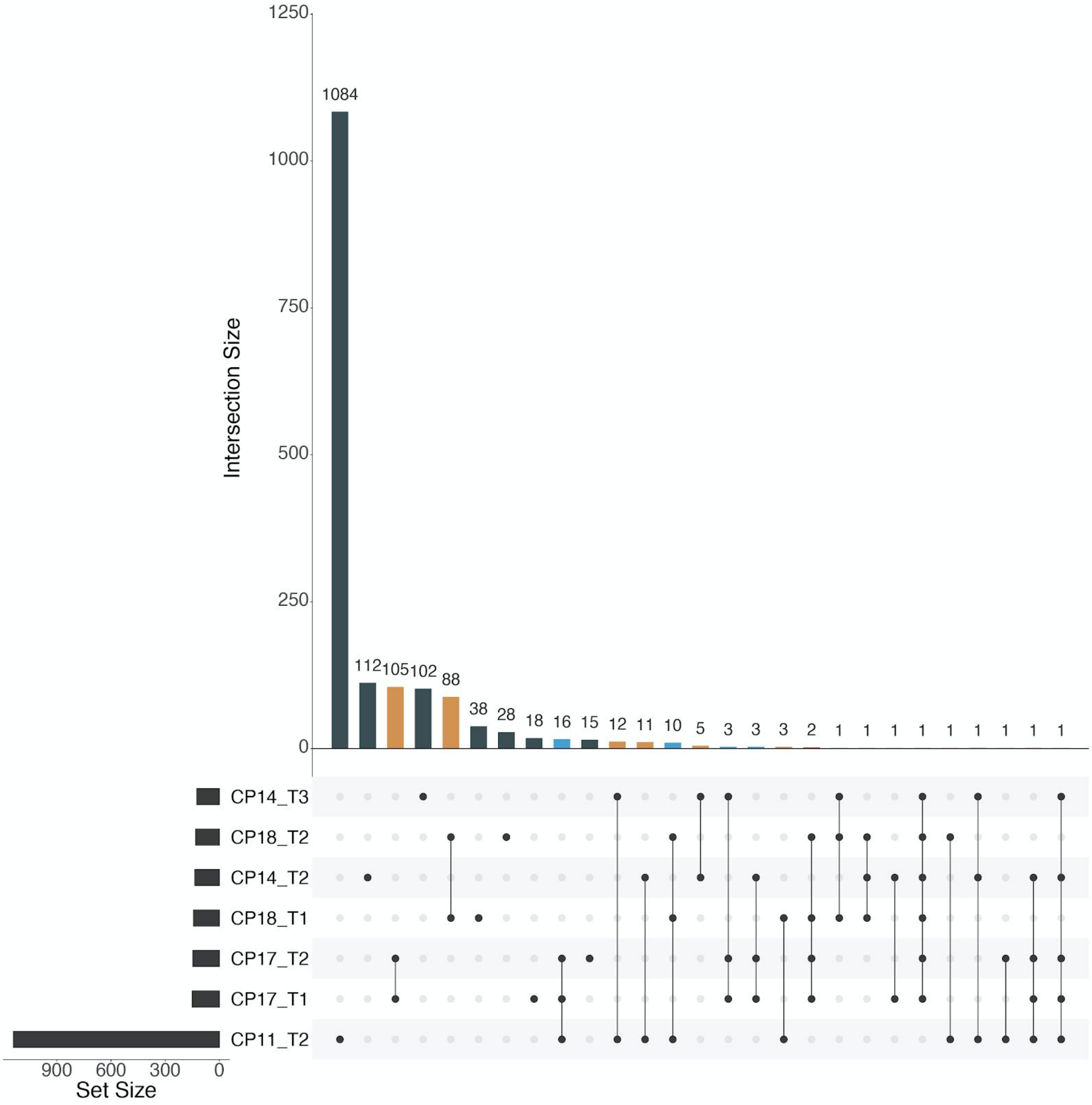

Supplementary Figure 15. Shared genes that contain mutations among the multiple-subclones primary tumor samples. A set is represented as a single dot or multiple dots that are connected by a line, and the bars on the top indicate the number of shared genes of each set. Thus, the figure starts from the left: no sharing, towards the right of shared among all samples involving multiple subclones.

Supplementary Note 1. Instruction of FastClone

1. Installation

   FastClone needs Python 3.5 or later version. It needs logbook, python-fire, scikit-learn, and pandas. To install the package, please use the following codes:

   git clone https://github.com/GuanLab/FastClone_GuanLab.git

   pip install FastClone_GuanLab/

(Please make sure you have the slash at the end, which forces pip to install from local directory, otherwise it will run into error)

   An alternative way is using pip to directly install FastClone:

   pip install fastclone-guanlab

2. Usage

   FastClone accepts either MuTect VCF + Battenberg format (specified in the DREAM SMC-Het Challenge) or PyClone format. The general format of the command line for running FastClone:

   fastclone load-[FILE_FORMAT] prop [FILE_NAME] [TUMOR_PURITY] solve [OUTPUT_PATHWAY]

   Here is an example to load a sample and infer:

   fastclone load-pyclone prop t1.tsv 0.8 solve ./fastclone_result

   （Please make sure t1.tsv is under your current directory）

Supplementary Software 1. FastClone

The code of FastClone also comes with this manuscript. You can also download it and install it by using the command <pip install FastClone_GuanLab/>.
