# Supplementary material for: FastClone is a probabilistic tool for deconvoluting tumor heterogeneity in bulk-sequencing samples
Source: Nat Commun. 2020 Sep 8;11:4469. doi: 10.1038/s41467-020-18169-2 (PMC7478963; doi:10.1038/s41467-020-18169-2)
Supplement: Supplementary file 3 — Description of Additional Supplementary Files [file 41467_2020_18169_MOESM3_ESM.pdf]

Description of Additional Supplementary Files

File Name: Supplementary Software 1

Description: FastClone. The code of FastClone also comes with this manuscript. You can also download it and install it by using the command <pip install FastClone_GuanLab/>.
