# Supplementary figures and images for: FastClone is a probabilistic tool for deconvoluting tumor heterogeneity in bulk-sequencing samples
Source: Nat Commun. 2020 Sep 8;11:4469. doi: 10.1038/s41467-020-18169-2 (PMC7478963; doi:10.1038/s41467-020-18169-2)

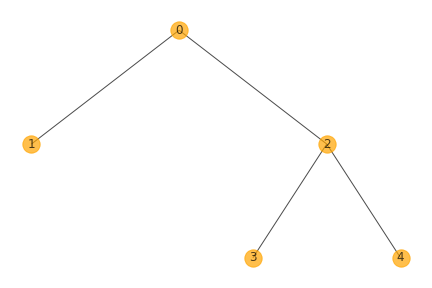

Supplement: Supplementary file 4 — Supplementary Software 1 [file 41467_2020_18169_MOESM4_ESM.zip › FastClone_GuanLab-master/example_phylogeny.png]
