# Supplementary material for: FastClone is a probabilistic tool for deconvoluting tumor heterogeneity in bulk-sequencing samples
Source: Nat Commun. 2020 Sep 8;11:4469. doi: 10.1038/s41467-020-18169-2 (PMC7478963; doi:10.1038/s41467-020-18169-2)
Supplement: Supplementary file 5 — Reporting Summary [file 41467_2020_18169_MOESM5_ESM.pdf]

nature research

Corresponding author(s): Yuanfang Guan
Last updated by author(s): Jul 7, 2020

# Reporting Summary

Nature Research wishes to improve the reproducibility of the work that we publish. This form provides structure for consistency and transparency in reporting. For further information on Nature Research policies, see our Editorial Policies and the Editorial Policy Checklist.

## Statistics

For all statistical analyses, confirm that the following items are present in the figure legend, table legend, main text, or Methods section.

| n/a | Confirmed | |
|---|---|---|
| ☐ | ☒ | The exact sample size (*n*) for each experimental group/condition, given as a discrete number and unit of measurement |
| ☐ | ☒ | A statement on whether measurements were taken from distinct samples or whether the same sample was measured repeatedly |
| ☐ | ☒ | The statistical test(s) used AND whether they are one- or two-sided<br>*Only common tests should be described solely by name; describe more complex techniques in the Methods section.* |
| ☐ | ☒ | A description of all covariates tested |
| ☐ | ☒ | A description of any assumptions or corrections, such as tests of normality and adjustment for multiple comparisons |
| ☐ | ☒ | A full description of the statistical parameters including central tendency (e.g. means) or other basic estimates (e.g. regression coefficient) AND variation (e.g. standard deviation) or associated estimates of uncertainty (e.g. confidence intervals) |
| ☐ | ☒ | For null hypothesis testing, the test statistic (e.g. $F$, $t$, $r$) with confidence intervals, effect sizes, degrees of freedom and $P$ value noted<br>*Give P values as exact values whenever suitable.* |
| ☐ | ☒ | For Bayesian analysis, information on the choice of priors and Markov chain Monte Carlo settings |
| ☐ | ☒ | For hierarchical and complex designs, identification of the appropriate level for tests and full reporting of outcomes |
| ☐ | ☒ | Estimates of effect sizes (e.g. Cohen's *d*, Pearson's *r*), indicating how they were calculated |

*Our web collection on statistics for biologists contains articles on many of the points above.*

## Software and code

Policy information about availability of computer code

| | |
|---|---|
| Data collection | MuTect v.1.1.4, VarScan somatic v.2.3.7, and Strelka v.1.0.14, Nexus Copy Number software v7.5, TAPS tool in Patchwork software library |
| Data analysis | FastClone [https://github.com/Guanlab/FastClone_Guanlab], PyClone v.0.13.1, Citup v.0.1.2 |

For manuscripts utilizing custom algorithms or software that are central to the research but not yet described in published literature, software must be made available to editors and reviewers. We strongly encourage code deposition in a community repository (e.g. GitHub). See the Nature Research guidelines for submitting code & software for further information.

## Data

Policy information about availability of data

All manuscripts must include a data availability statement. This statement should provide the following information, where applicable:

- Accession codes, unique identifiers, or web links for publicly available datasets
- A list of figures that have associated raw data
- A description of any restrictions on data availability

-The simulated tumor benchmark data are provided by the SMC Tumor Heterogeneity Challenge. Data are available at [https://guanfiles.dcmb.med.umich.edu/FastClone/]. The colon data we
analyzed in the paper are available at [https://github.com/GuanLab/FastClone_GuanLab/tree/master/colon_data]. The original colon tumor variant data are available at the European Variation
Archive (EVA) [https://www.ebi.ac.uk/eva/?eva-study=PRJEB23791], and the SNP array data are available at GEO [https://www.ncbi.nlm.nih.gov/geo/query/acc.cgi?acc=GSE107225].

# Field-specific reporting

Please select the one below that is the best fit for your research. If you are not sure, read the appropriate sections before making your selection.

☒ Life sciences ☐ Behavioural & social sciences ☐ Ecological, evolutionary & environmental sciences

For a reference copy of the document with all sections, see nature.com/documents/nr-reporting-summary-flat.pdf

# Life sciences study design

All studies must disclose on these points even when the disclosure is negative.

| | |
|---|---|
| Sample size | 15 tumors for colon cancer and 8 for simulated data. We did not do sample size calculation. 8 simulated data were provided directly by the organizer of SMC challenge, and 15 tumors samples are determined by how many available samples the wet lab have, but we think around twice as much as the simulated sample size is appropriate for testing the performance of our software on real data. |
| Data exclusions | CP11 T1 is excluded as deep sequencing failed for this sample. |
| Replication | binary reproducible |
| Randomization | Not applicable. Our experiment does not include a comparison between treatment and placebo group. |
| Blinding | Not applicable. Our experiment does not include a comparison between treatment and placebo group. |

# Reporting for specific materials, systems and methods

We require information from authors about some types of materials, experimental systems and methods used in many studies. Here, indicate whether each material, system or method listed is relevant to your study. If you are not sure if a list item applies to your research, read the appropriate section before selecting a response.

## Materials & experimental systems

| n/a | Involved in the study | |
|---|---|---|
| ☒ | ☐ | Antibodies |
| ☒ | ☐ | Eukaryotic cell lines |
| ☒ | ☐ | Palaeontology and archaeology |
| ☒ | ☐ | Animals and other organisms |
| ☒ | ☐ | Human research participants |
| ☒ | ☐ | Clinical data |
| ☒ | ☐ | Dual use research of concern |

## Methods

| n/a | Involved in the study | |
|---|---|---|
| ☒ | ☐ | ChIP-seq |
| ☒ | ☐ | Flow cytometry |
| ☒ | ☐ | MRI-based neuroimaging |
